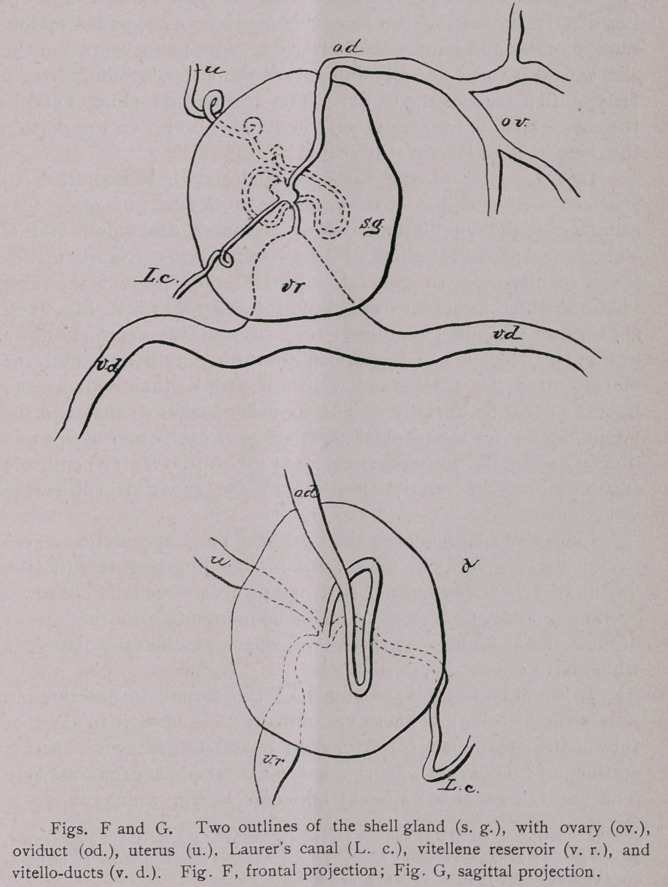# The Anatomy of the Large American Fluke (*Fasciola Magna*)

**Published:** 1894-09

**Authors:** Charles Wardell Stiles

**Affiliations:** Zoȯlogist, Bureau of Animal Industry


### THE JOURNAL
OF
### COMPARATIVE MEDICINE AND VETERINARY ARCHIVES.

VOL. XV. APRIL, 1894. NO. 4.

### THE ANATOMY OF THE LARGE AMERICAN FLUKE (*FASCIOLA MAGNA*), AND A COMPARISON WITH OTHER SPECIES OF THE GENUS FASCIOLA, S.ST.

BY CHAS. WARDELL STILES, PH.D.,
*Zoölogist, Bureau of Animal Industry.*

### CONTAINING ALSO A LIST OF THE CHIEF EPIZOÖTICS OF FASCIOLIASIS (DISTOMATOSIS) AND A BIBLIOGRAPHY OF *FASCIOLA HEPATICA.*

BY ALBERT HASSALL, M.R.C.V.S.

(Continued from page 178.)

### MORPHOLOGY.

*External appearance* —When examined immediately after being taken from the liver or lungs, *F. magna* is usually of a fleshy tinge, hence Hassall's specific name *carnosa*, occasionally more of a slate color, its dorsal surface being lighter than its ventral surface. In form it resembles a flat fish or a leaf, to a certain extent. In the general contour (Plate I, Figs. 2 and 3, and Plate II) of the body there is considerable difference between *F. magna* and *F. hepatica*. Although in both forms the worm presents a conical anterior portion limited posteriorly by the acetabulum (ventral sucker), and a posterior portion which is flatter and broader, the division between the two regions is much less distinct in *F. magna* than in *F. hepatica*. In fact in most specimens of the former species, the conical form of the anterior portion disappears entirely, the two shoulders so common in *F. hepatica* at the height of the acetabulum, being replaced by a convex margin, so that there is no marginal boundary between the anterior conical and the posterior flat portion. Specimens are occasionally found which agree perfectly with Cobbold's figure of *F. Jacksoni*. The portion in front of the acetabulum is lighter than the portion posterior to the same. The darker tint of the posterior portion is due to the intestines and genital organs.

The contour (Plate II) of the worm varies greatly according to the state of contraction. In general, however, it may be said that the broadest portion of the worm is the middle third of the body. The anterior third decreases in breadth quite rapidly and the anterior extremity is more or less bluntly pointed. The posterior third decreases in breadth much more gradually and the posterior extremity is quite bluntly rounded. In the case of *F. hepatica* on the other hand, the posterior fourth of the body becomes quite narrow in comparison with the middle of the body and the posterior extremity is bluntly pointed. Although the lateral margins of the middle half of the worm are usually convex, specimens are occasionally found, the sides of which are almost parallel, thus agreeing with *F. gigantea* in form.

The body is thickest (2–4.5 mm.) at about the position of the ventral sucker or just behind this organ. From this region the body grades quite rapidly and regularly to the mouth; posteriorly and laterally the body diminishes in thickness more gradually, at the margin becoming very thin.

Specimens sent to us by Prof. Sonsino, Italy, measured as follows:

| Length. | Greatest Breadth. | Gen. pore from Anterior Extremity. | Acetabulum from Anterior Extremity. |
|---|---|---|---|
| mm. | mm. | mm. | mm. |
| 72 | 28 | 4.75 | 8 |
| 60 | 18 | 3.5 | 5 |
| 59 | 24 | 4 | 7 |
| 55 | 24 | 4 | 6.5 |
| 57 | 22 | 4.5 | 7.5 |
| 48 | 21 | 3.75 | 7 |
| 34 | 20 | 3.5 | 5.5 |
| 27 | 10 | 3.5 | 5 |

In his article ('90) Sonsino records a specimen 100 mm. long.

PLATE II.—*F. magna.*

Sixteen specimens of different size, contour and stages of contraction. All natural size. The mouth, genital pore and acetabulum are visible.

Specimens taken in this country presented the following measurements (made on preserved material):

| Length. | Greatest Breadth. | Gen. pore from Anterior Extremity. | Acetabulum from Anterior Extremity. |
|---|---|---|---|
| mm. | mm. | mm. | mm. |
| 23.5 | 19 | 1.5 | 3 |
| 26 | 11.5 | 2 | 3.5 |
| 26 | 17 | 2 | 4 |
| 32.5 | 17.5 | 3.5 | 6 |
| 34.5 | 19.5 | 2.5 | 5 |
| 35 | 14 | 3.5 | 7 |
| 35.5 | 18 | 2.5 | 5 |
| 37 | 16.5 | 2 | 3.5 |
| 39 | 24 | 3.5 | 7 |
| 42 | 13.5 | 2.5 | 5 |
| 42 | 14 | 2.5 | 5 |
| 45 | 23 | 2 | 4.5 |
| 49 | 17.5 | 3.5 | 7 |
| 56 | 22.5 | 4.5 | 9 |
| 59.5 | 25.5 | 3.5 | 6 |
| 73 | 26 | 4 | 8 |

Measurements by Francis are given as 30–73 mm. in length by 20–30 mm. in breadth. He found some specimens which were 8 mm. long by 4 mm. broad.

At the anterior extremity is the deep oral sucker, tending slightly ventrally; 1.5 mm.–4.75 mm. caudad of the same, in the median line, lies the genital pore, from which the cirrus frequently extrudes; 3–9 mm. (4–5 mm. after Francis) caudad of the mouth, in the ventral median line is the acetabulum. The latter is sometimes larger (2.5 mm.) than the oral sucker (1.5 mm ). Its opening varies between an equilateral triangle—the base anterior, the apex posterior—and a circle. Near the posterior margin of the body in the dorsal median line is found a small opening, the pore of the excretory canal.

*Anatomy.*—Almost the entire anatomy can be made out upon unpreserved specimens which are allowed to macerate a few days in water or in very weak alcohol. Such specimens are drawn on Plate I, Figs. 2 and 3, the digestive organs and sexual organs being shown in separate figures to avoid confusion.

It will be seen that the oral sucker is followed by a pharyngeal bulb, this in turn, by an œsophagus, which is generally from 1½–3 times as long as the bulb. The œsophagus then branches at iright angles like ⊥ into the two primary intestinal cæca mmediately anterior to the genital pore. These primary branches, extend anteriorly nearly to the mouth, and posteriorly, one on each side of the median line, to the posterior extremity of the animal. Each intestinal branch sends off secondary branches, both towards the median line and the lateral margins. These secondary branches then give off smaller branches both anteriorly and posteriorly (Fig. A. and Plate I, Fig. 2). It will, however, be noticed that a space is frequently left in the median line which is not covered by these branches. I have counted from 16 to 25 lateral cæca and about the same number of median branches.

Fig. A. Outline of oral sucker, pharynx, œsophagus, anterior portion of right intestinal cæcum with its numerous branches, and acetabulum.

*Male genitalia.*—The cirrus (Plate I, Fig. 3) is generally quite prominent. The vasa deferentia are difficult to see. Two branched testicles lie, one on each side of the median line, in about the middle of the body, the right testicle being slightly posterior to the left testicle. In one specimen (Plate I, Fig. 3) examined, these testicles were entirely distinct from each other, a space remaining unoccupied by them in the median line. As a general rule, however, the right and left testicles cannot be distinctly separated from each other, for branches of each extend into the field of the other.

*Female genitalia.*—Vitellogene glands (Plate I, Fig. 3) occupy almost the entire marginal field, with the exception of the anterior conical portion of the body. The vitello-ducts can be traced for a short distance, one running from each side transversely towards the median line, at about the posterior border of the anterior third of the body. In the median line they meet in a common reservoir and enter the "shell-gland," which also receives the oviduct. The ovary is much whiter than the other organs, is quite ramified and lies, in most cases that I have examined at least, on the right side of the body. From the shell-gland, the uterus can be traced to a point near the acetabulum.

*Nerves.*—Two ventral longitudinal nerves (Plate I, Fig. 3) can be seen running from the oral sucker, and diverging posteriorly. They can be traced to about the middle of the body.

It will be seen from this short description that the organization agrees very well with that of *F. hepatica.* In order to obtain further details one must have recourse to stained specimens, both press-preparations and microtome sections.

*Cuticle.*—The entire surface of the worm is covered with a cuticle, which extends into the various apertures (see below) of the body; it becomes much thicker on the ventral surface, and in the thick portions shows the palisade-like structure mentioned by Leuckart in *F. hepatica* (l. c., p. 186). It is pierced by numerous apertures, 9 $\mu$ or more in diameter, through each of which extrudes a spine (Plate I, Fig. 7). The spines are rather conical in shape, slightly curved, 42 $\mu$–52 $\mu$ long, 9 $\mu$–12 $\mu$ in diameter at the base; they extend down through the cuticle to the muscular layer, and possess a rather blunt apex which is directed caudad.

The *acetabulum* is 1.6 to 2.5 mm. in diameter; its muscular wall is 0.48 mm. in thickness. Its general structure agrees with that of *F. hepatica*.

The *muscles* lying directly under the investing cuticle of the body also agree with those described for *F. hepatica*, i. e., 1, the outer layer is composed of circular fibres; 2, the next layer of longitudinal fibres, and 3, the inner layer of a double set of diagonal fibres.

The only point I will mention in connection with the *parenchyma*, which appears to be the same in all allied forms, is that I have found a number of large cells near the surface of the body, which agree in appearance with the cells found by various authors in the acetabulum, oral sucker and pharyngeal bulb. Whether they really are identical in origin, I am unable to state; as to the function of these cells see below.

*Digestive tract.*—The oral sucker (Figs. B and C) measures 0.96 mm.–1.5 mm. in transverse section. Its longitudinal section measures 0.48 mm.; the muscular wall is 0.21 mm. thick. It is composed of the following:

1. A cuticle which lines its cavity.

2. A layer of muscular fibres which run parallel to its concave surface; in transverse section of the body, longitudinal sections of these fibres are seen, while in frontal sections of the body, transverse sections of the muscles come into view. This layer becomes much thicker nearer the pharynx.

3. A layer of muscular fibres of which the transverse sections

Fig B. Longitudinal section of oral sucker, post-buccal ring and pharynx.

Fig. C. Transverse section of oral sucker. For description see text appear on transverse sections of the body, and longitudinal sections in frontal sections of the body. They too become heavier near the pharynx. Layers 2 and 3, running at right angles to each other, are evidently Leuckart's *æquatorial* and *meridional fibres.*

4. On each side of the median portion of the sucker there is quite a highly developed system of muscular fibres which are seen in cross section on frontal sections of the body and in longitudinal section on transverse and sagittal sections of the worm.

These fibres extend from the lateral margins (each side) around through about the middle of the muscular wall in such a way as to form almost a circular layer on each lateral third of the sucker, the median third of the sucker being free from them. The fibres then run dorsally, pierce the dorsal boundary of the sucker, scatter through the surrounding parenchyma of the body and insert on the dorsal portion of the worm. They are not evenly distributed in the sucker, but lie in bundles.

At the boundary between the sucker and the parenchyma of the body, are found two layers of muscles which cross each other at right angles.

5. Nearest the parenchyma is a layer which appears in transverse section on frontal sections, but *in toto* on transverse sections, the continuation of 2.

6. A layer inside of these (*i. e.*, nearer the opening of the sucker), which is a continuation of layer 3. The fibres appear *in toto* on frontal sections and as transverse sections in transverse sections of the sucker.

7. Extending between the two borders of the sucker are numerous radial muscles (*i. e.*, at about right angles to the cuticle).

Nerve-fibres also enter the sucker, and scattered through the latter are numerous small nuclei, as well as larger round and very peculiar nuclei, which various authors look upon as ganglion cells (Leuckart and others), or as connective tissue nuclei (Looss). Preparations stained with acid carmine speak decidedly in favor of the interpretation given by Looss.

As stated above, I have found in *F. magna* a number of nuclei very similar, perhaps identical with these in the parenchyma of the body near the muscular wall.

Leuckart's description of the pharynx of *F. hepatica* applies in a general way quite well to this species also. There are, however, some details in regard to which the description cannot be applied. Leuckart states that the pharynx is not entirely separated from the buccal sucker, but that the anterior end of the former is connected with the latter for a short distance (l. c., fig. 101). I, however, find that both in this species and in *F. hepatica* the two organs are distinctly separated by the* post-buccal cavity (*Vorhöhle of Leuckart*). In transverse sections it frequently appears as if the pharynx and oral sucker were connected, but in frontal and sagittal sections the circular post-buccal cavity can always (*i. e.*, in my preparations) be distinctly seen separating the two. The pharynx of *F. magna* measures 0.8 mm. long. It is made up of the following:

1. A cuticle, in which remains of nuclei are occasionally found.

2. Numerous radial muscles which make up the greater part of the organ.

3. A layer of circular muscles next to the cuticle, seen in transverse section on frontal sections of the worm, *in toto* on transverse sections of the parasite. This layer is about 8 $\mu$ thick, except in the posterior portion of the bulb, where it increases to about 16 $\mu$.

4. Near the periphery of the pharynx is a layer of circular muscles. In the lateral portion of the bulb, these muscles are very close to the periphery, but they come quite close to the lumen on the dorsal and ventral surfaces.

5. Exterior to the circular muscles is found a heavy layer of longitudinal muscles. This layer appears thicker in the anterior portion than in the posterior portion.

6. There is a reticulum of parenchymatic appearance, extending throughout the entire organ, and here and there are seen large (42 $\mu$) cells (nucleus 16 $\mu$, nucleolus 6 $\mu$), such as were mentioned in connection with the oral sucker.

The muscular wall of the pharynx is about 0.18 mm. thick; its dorso-ventral diameter is 0.770 mm., its lateral diameter 0.450 mm. These measurements are subject to a slight variation due to individual variation and contraction.

The entire bulb is surrounded by the *protractor pharyngis*, a series of muscular fibres which extend from the posterior end of the pharynx to the posterior portion of the post-buccal cavity, some of the fibres inserting in the oral sucker.

*Post-buccal cavity* (Fig. B).—Between the oral sucker and the pharynx is found a post-buccal ring. This is circular in form, is quite shallow at the lateral portion of the pharynx, but somewhat

---

* In all probability this difference between Leuckart's results and mine are, I believe, to be explained by slight individual variations.

deeper on its dorsal aspect and still deeper on its ventral aspect.*

The cuticle at the deepest portion of this ring is much thicker than at the upper portion, and has very much the appearance of an epithelium similar to that in the intestine. This is due to a splitting of the cuticle.

*Œsophagus.*—Judging from the published figures of *F. hepatica* authors are quite generally agreed that the œsophagus of that species is extremely short. In most cases it is drawn about equal in length to the pharynx, frequently shorter than the pharynx. In my own preparations it is generally equal to or shorter than the pharynx, but in a few cases it is longer than that organ. In *F. magna*, on the other hand, the œsophagus (Fig. A) is from 1½ to 3 times as long as the pharynx, averaging about 1.4 mm. long. It is 0.26 mm. in diameter. Its component parts are: 1, a lining cuticle; 2, a layer of circular muscle-fibres, and 3, a layer of longitudinal fibres. The layer of circular muscles is thickest very close to the pharynx. Posteriorly, the œsophagus branches, sending a prolongation at nearly right angles to each of the intestines.

Fig. D. Transverse section of body to show the intestines.

*Intestine.*—The intestine proper (Fig. A, and Plate I, Fig. 2) bears a close resemblance to the intestine of *F. hepatica.* The two longitudinal canals give off numerous branches as described above. On transverse sections (Fig. D) it will be noticed that

*This is another point where my results differ from Leuckart's. Leuckart states that the post-buccal ring is deeper dorsally than ventrally, and that a pouch is present ventrally. Mehlis (according to Leuckart) considered this pouch as a portion of the post-buccal ring, but Leuckart himself looks upon it as an independent organ. Of some ten different series of sections of *F. hepatica* and *F. magna* only one of them seem to support Leuckart's view, and even the pictures yielded by this series permitted another interpretation. The remaining series certainly supported the view advanced by Mehlis. That there should be this difference between Leuckart's description of *F. hepatica* and mine of *F. magna* is not so strange, but that my series of *F. hepatica* do not support Leuckart's description seems rather peculiar to me. The matter finally resolves itself to the point that either I and Mehlis are in error, or Leuckart is in error, in the interpretation of our slides, or that here too there may be an individual variation.

the various tubes have a semi-lunar arrangement, the concavity being directed ventrad. The histology agrees with that of *F. hepatica.* The high columnar epithelium bounds the lumen, the cells resting upon a thin membrane; this, in turn, is surrounded by longitudinal and circular muscle-fibres. The arrangement of these fibres does not appear to be constant. While the order described by Leuckart, circular fibres between two layers of longitudinal fibres, is frequently distinguishable, it not infrequently occurs that the circular fibres are outermost.

*Nervous system.*—The nervous system (Fig. E) does not differ materially from that of *F. hepatica.* On each side of the anterior portion of the pharynx there is situated a large ganglionic mass; the two ganglia are connected by a commissure crossing the pharynx dorsally. From the anterior point of these ganglia a ventral

Fig. E. Nervous system, for description see text.

nerve (1) runs to the oral sucker. Just dorsally of these nerves a pair of nerves (2) branches to the anterior portion above the sucker. The third pair of nerves (3) extends laterally and anteriorly. The fourth pair (4) extends laterally and posteriorly and then branches. Immediately dorsal of this pair, a longitudinal dorsal nerve (5) extends posteriorly to about the position of the ventral sucker. Median and ventrally of this, a large ventral nerve (6) extends posteriorly and gives off branches both towards the median line and towards the sides of the animal. From a point anterior and median of the origin of these ventral nerves a seventh (7) pair of nerves runs medio-ventrally to a position ventrad and very slightly posterior to the pharynx, where they unite in a common ganglionic mass. The eighth pair of nerves (8) is anterior to this point and supplies the pharynx.

*Genital organs.*—The genital pore, as stated above, lies immediately ventrad and posterior to the bifurcation of the œsophagus. On the right is the cirrus, on the left the vulva.

*Male genitalia.*—The cirrus, as is the case with *F. hepatica*, is sometimes extruded and resembles a narrow tongue in form, sometimes inverted in the cirrus-pouch. Its lumen empties into a ductus ejaculatorius which widens into the vesicula seminalis, into the posterior end of which the two vasa deferentia open. The latter then extend caudad passing dorsally of the uterus. When the uterus becomes longer some of its loops extend around dorsally of the vas deferens.

In nearly all published figures of *F. hepatica* the vasa deferentia cross the vitello-ducts dorsally, but in all of my preparations, both of *F. hepatica* and *F. magna*, the vasa deferentia cross the vitello-ducts ventrally. The vas deferens on the same side of the body as the ovary runs dorsally of the latter.

The vas deferens of the right side extends slightly further caudad than that of the left side. Both vasa deferentia branch into the testicular canals which give off numerous secondary canals. These testicular canals cross each other, forming an irregular network. In many places they seem to anastomose, in other places it is difficult to decide whether they cross each other or anastomose. The testicles have an extreme ventral position.

*Histology.*—The inverted cirrus shows the following structure: 1, canal, 21 $\mu$ in diameter; 2, cuticle, 12 $\mu$ thick; 3, heavy layer of circular muscles; 4, longitudinal muscle-fibres. The narrow portion of the ductus ejaculatorius is lined with a thin cuticle under which lies an epithelium, followed by circular and longitudinal muscles. In the vesicula seminalis the cuticle almost disappears. While the ends of the epithelial cells of the narrower portion are very irregular and extend down into the muscular layer, in the vesicula seminalis the epithelial cells become more regular in outline. The muscular portion becomes thinner. The vasa deferentia empty into the vesicula seminalis sometimes within the cirrus-pouch, sometimes slightly caudad of the same. In the muscular wall of the cirrus-pouch can be distinguished circular, longitudinal and irregularly diagonal fibres. The space between the muscular wall and the inner canal (v. s., etc.) is occupied by gland-cells and a connective tissue.

*Female Genitalia.*—The vulva is situated at the left of the cirrus; it is lined by an invagination of the cuticle, this in turn being surrounded by circular and longitudinal muscles. A canal can be traced from the vulva to the middle of the shell-gland, presenting a somewhat different appearance in its different portions. The first portion is generally rather narrow and presents a cuticle, heavy circular muscles and longitudinal muscles. This serves as the vagina, sometimes it contains eggs and is very broad.

Figs. F and G. Two outlines of the shell gland (s. g.), with ovary (ov.), oviduct (od.), uterus (u.), Laurer's canal (L. c.), vitellene reservoir (v. r.), and vitello-ducts (v. d.). Fig. F, frontal projection; Fig. G, sagittal projection.

The vagina extends on the left of the cirrus-pouch, gradually coming to lie ventrally of it. As transverse sections are followed caudad, it is noticed that the lining of the canal becomes raised at a number of points, the raised portion resembling columnar cells, but possessing no nuclei. At a point over the middle of the acetabulum the lining becomes distinctly cellular, the nuclei coming plainly into view.

From this point on, the uterus extends in a number of loops posteriorly, possessing the same histology, *i. e.*, cylinder epithelium. circular and longitudinal muscles. At a point ventral of the shell-gland it turns dorsally and enters the latter, winding irregularly, until it reaches the center. The last portion (Figs. F–G) in the shell-gland, is frequently very much narrower. In this portion the circular muscles are still visible.

In the center of the so-called shell-gland, is a short canal (*Centralraum*) running dorso-ventrally, and into this open four canals, *i. e.*, the vitello-duct, oviduct, Laurer's canal and uterus. The topographical relations of these canals vary slightly in different specimens. In general, however, they present the same relations which Leuckart has figured for *F. hepatica* (l.c., Fig. 113). On sagittal sections (combined) they present the relation shown in Fig. G. Here it will be noticed that the vitello-duct and the uterus enter the shell-gland ventrally, while the oviduct and Laurer's canal lie dorsally. The oviduct, Laurer's canal, and the vitello-duct enter the central chamber near each other at one end (dorsal), while the uterus extends from the other (ventral) end. As stated above, the relative position of the canals is subject to variation.

Laurer's canal has been the subject of much speculation on the part of various workers, most authors homologizing it with the vagina of **Bothriocephalus.* According to a view recently advanced by Looss, however, Laurer's canal is homologous with the uterus of cestodes. Looss' interpretation seems to be the best one which has yet been suggested.

It would be beyond the object of this article to describe minutely the histology of these various organs. Suffice it to say that the central chamber (*Centralraum*) possesses a very distinct epithelium (nuclei 3–4 $\mu$) with a basement membrane (or muscles?). The Laurer's canal has a heavy cuticular lining, which varies in

*For a discussion of this subject vide: Leuckart, Die Parasiten des Menschen I. 2. pp. 55–60 ; Braun's Vermes ; Looss, Ist der Laurer's sche Kanal der Trematoden eine Vagina ? (C. f. B. u. P. XIII. pp. 808–819.)

thickness according to the diameter of the lumen. This cuticle is surrounded by a thin circular layer which is possibly muscular in nature; this, in turn, by a mass of tissue in which nuclei are more or less regularly distributed. In the lower portion of the uterus, one finds ova with the forming egg-shells, vitellene cells, and a mass of yellowish matter. Authors generally assume that this yellowish matter forms the shell and is itself the product of the shell-gland (Leuckart). Leuckart adds (l. c., p. 232): "Ich meinerseits bin gleichfalls der Ansicht, dass diese Massen den Drüssenzellen entstammen, glaube aber, die gelbe Farbe derselben auf die Dotterzellen zurückführen zu müssen, welche nach dem Uebertritt in die weiblichen Leitungswege die in sie eingelagerten gelben Körner mehr oder minder vollständig verlieren und nach aussen hervortreten lassen, so dass diese dann mit dem an sich farblosen Secrete der Schalendrüsen verschmelzen können. So viel ist jedenfalls gewiss, dass der Inhalt der Schalendrüse niemals gelb gefärbt ist."

My study of *F. hepatica* and *F. magna* would lead me to go even a step further than Leuckart. I believe that altogether too much importance has been attached to the shell-gland and too little to the vitellogene glands, in regard to the formation of the egg-shell. In fact, I believe that almost the entire material for the egg-shell is furnished by the vitellene cells. In support of this view I will present the following observations. The vitellene cells, while still in the vitellogene glands, in the vitello-ducts and the vitello-reservoir, are charged with small and large yellowish globules. As the cells ascend from the vitellogene glands to the central chamber of the shell-gland, either many of these globules must be excreted from the cells or many of the cells must perish, thus setting the globules free, for the vitello-ducts, etc., contain numerous smaller or larger globules of yellowish matter, which are exactly similar to those found in the lower portion of the uterus during the process of formation of the egg-shells. It must be remembered that these free globules are numerous even before the vitellene cells have entered the shell-gland; in fact, many may be found in the beginning of the vitello-duct or even in the vitellogene glands.

As the vitellene cells join the ova, the yellowish globules disappear from the former almost entirely and become more numerous in the lumen of the uterus, and all gradations can be found, between a minute yellowish globule and an entire egg-shell, so that it seems to me certain that the yellowish matter formed by the vitellene cells enters directly into the composition of the egg-shell.

Whatever secretion is formed by the shell-gland is certainly colorless, as Leuckart states.

If this interpretation is correct, then the function of the shell-gland would probably be to secrete a fluid which would act upon the yellowish particles and render them more plastic.

In regard to the vitellogene glands, it is worthy of note that while they extend both dorsally and ventrally of the intestines in *F. hepatica*, in every specimen of *F. magna* which I have examined, they are confined almost entirely to the ventral side of the intestines, although here and there a branch extends dorsally.

*Excretory apparatus.*—The excretory apparatus agrees essentially with that of *F. hepatica.* It runs dorsally of the intestines.

*Ova.*—(Plate I, Figs. 4–5). The eggs of *F. magna* can hardly be distinguished from those of *F. hepatica;* in general, however, they are slightly larger than the measurements given for *F. hepatica*, as will be seen by the following table:

| *F. magna.* | | *F. hepatica.* | |
|---|---|---|---|
| Long. | Broad. | Long. | Broad. |
| mm. | mm. | mm. | mm. |
| 0.109–0.168 | 0.075–0.096 | 0.105–0.145<br>0.13–0.14<br>0.13–0.172 | 0.066–0.090 (R. Blanchard)<br>0.075–0.09 (R. Leuckart)<br>0.072–0.08 *(Stiles) |

The *Miracidium* (Plate I, Fig. 6).—On several different occasions I have raised the embryos of this species from eggs, sent from Chicago by Dr. Melvin, from Arkansas by Dr. Dinwiddie, or collected at Washington by Dr. Hassall. Like the miracidium of *F. hepatica* it is covered with a ciliated epithelium. On the anterior end is found a papilla in which an opening is perfectly visible. This opening leads into a thin string of tissue, evidently a rudimentary œsophagus, ending in a double-lobed body which, from analogy with *F. hepatica*, represents the rudimentary intestine. Immediately posterior to this, is situated the ganglionic mass with the two cup-shaped eyes. In the posterior portion of the body a number of germ-cells can be distinguished. All of these observations were made upon living specimens, and as the

* American specimens.

similarity with the miracidium of *F. hepatica* is so striking, it was not deemed necessary—for the purpose of this paper—to prepare microtome sections of this stage.

The ciliated organism swims around rapidly in the water, changing its form as quickly and as much as does the miracidium of *F. hepatica.* A detailed description of these movements would simply be a repetition of the description of *F. hepatica* given by Leuckart and Thomas. The measurements naturally change with the change of form; in general, however, the miracidium agrees in size very well with the miracidium of *F. hepatica*, *i.e.*, 0.15 mm. long by 0.04 mm. broad, the posterior extremity being narrower than the anterior.

*Later stages.*—From the similarity between the anatomy of *F. hepatica* and *F. magna* it may be confidently expected that the life-history of the large American fluke will be very similar to that of *F. hepatica*, as determined by the investigations of Leuckart and Thomas. Experiments to raise the different stages are now in progress in the laboratory, and it is hoped that some positive results may soon be reported. It is, however, difficult to procure the proper intermediate host of the parasite so far from the locality where the organism lives, and it may be necessary to carry on the experiments in Texas or Arkansas, before the entire life-cycle can be demonstrated.

### Specific Diagnosis.

*F. magna* (Bassi, 1875) Stiles, 1894.—The flesh-colored body is much larger and thicker than *F. hepatica*, measuring 23-100 mm. long by 11–26 mm. broad by 2–4.5 mm. thick. The anterior portion is not so distinctly separated from the posterior portion. The margin is more convex and the posterior extremity is bluntly rounded. The ventral surface is convex and much darker than the flat dorsal surface. The general organization is very similar to that of *F. hepatica*, but the œsophagus is generally longer in proportion to the pharynx, the intestines are more ramified, and the vitellogene glands are confined almost entirely to the ventral side of the intestines. Eggs measure 109–168 $\mu$ long by 75–96 $\mu$ broad.

*To be continued.*

## Figures and Tables

**Figure f1:**
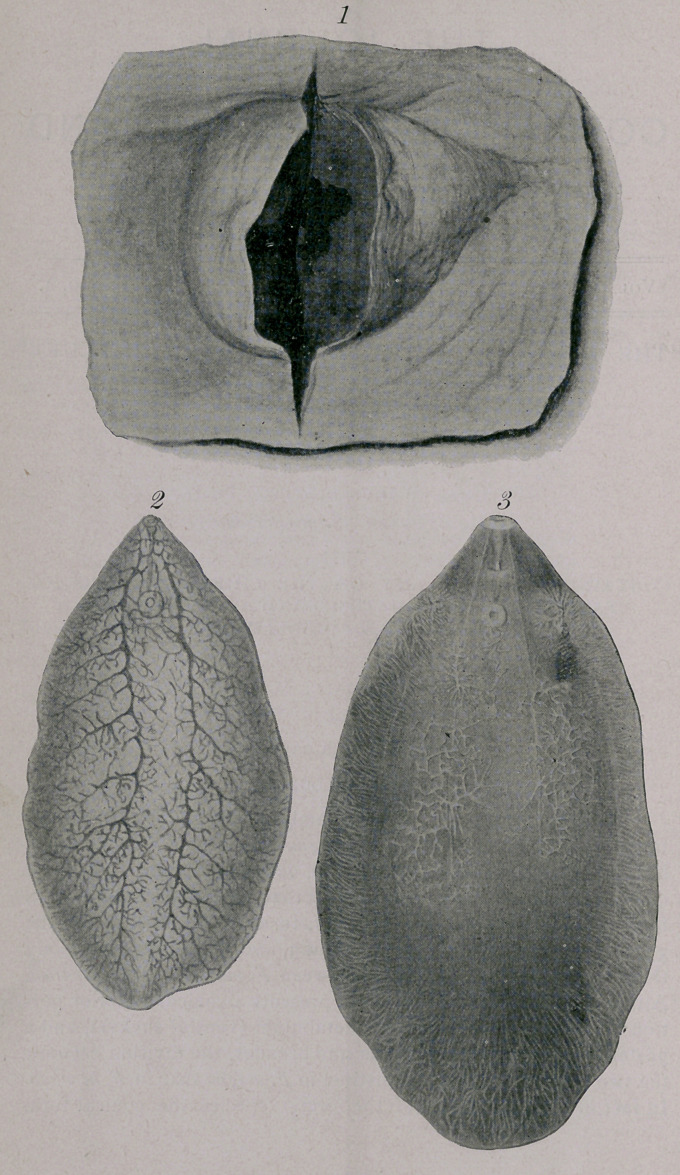


**Plate I. f2:**
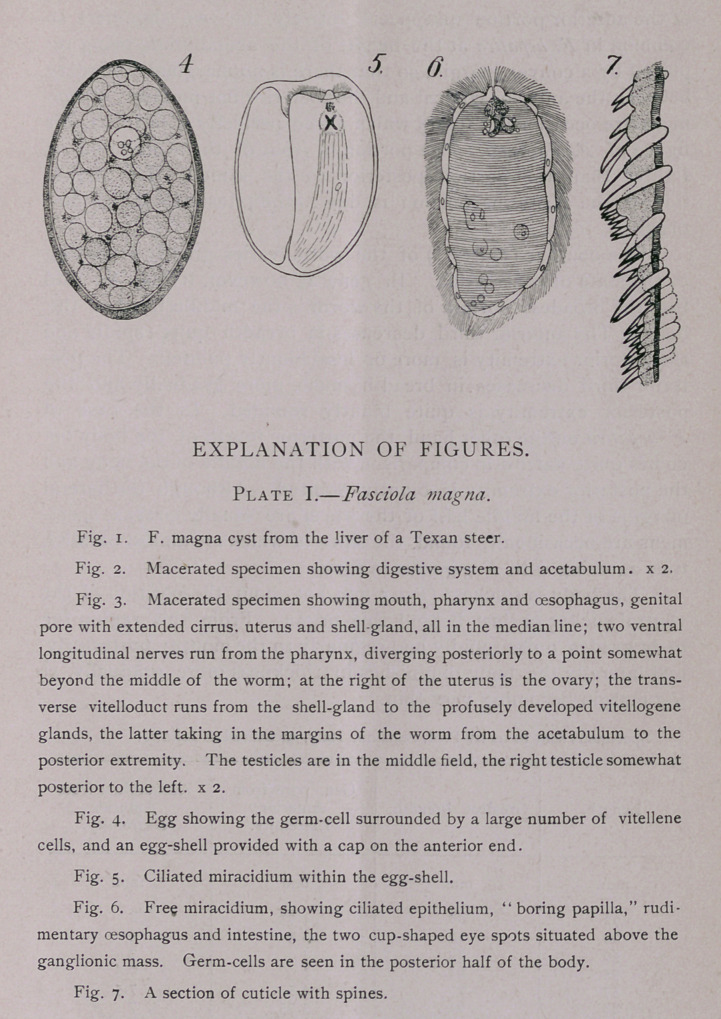


**Plate II. f3:**
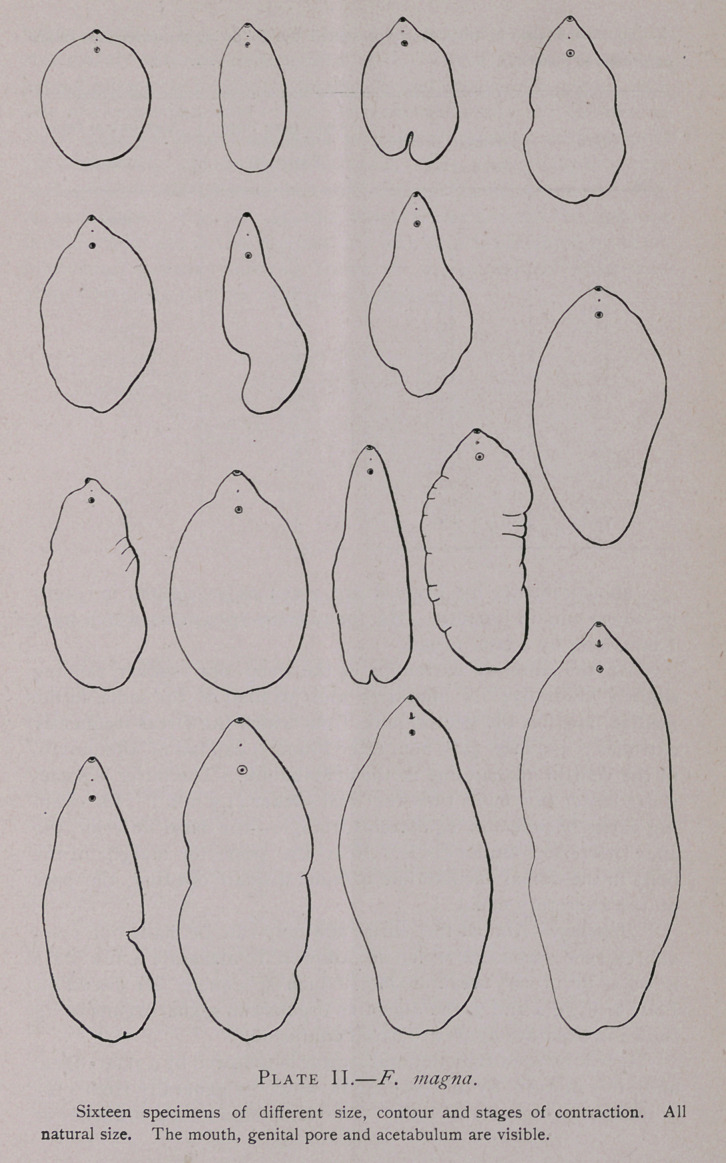


**Fig. A. f4:**
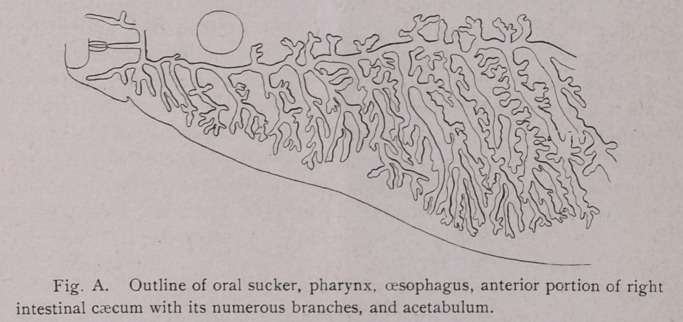


**Fig B. f5:**
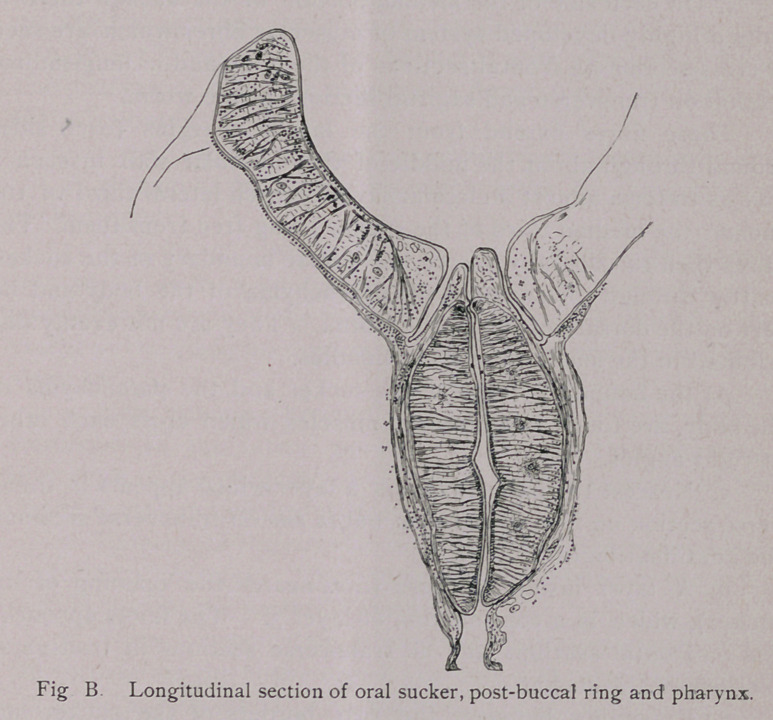


**Fig. C. f6:**
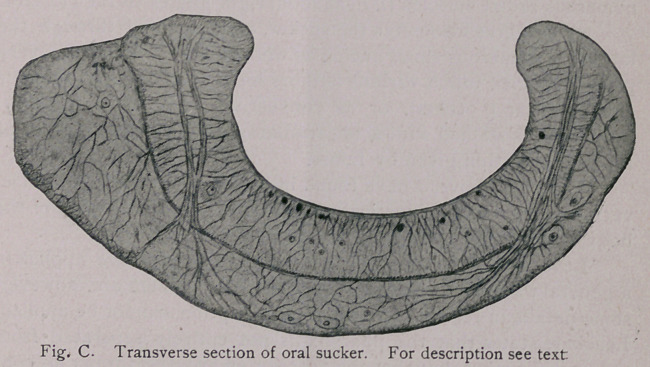


**Fig. D. f7:**
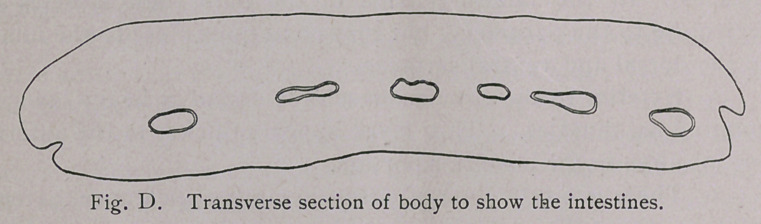


**Fig. E. f8:**
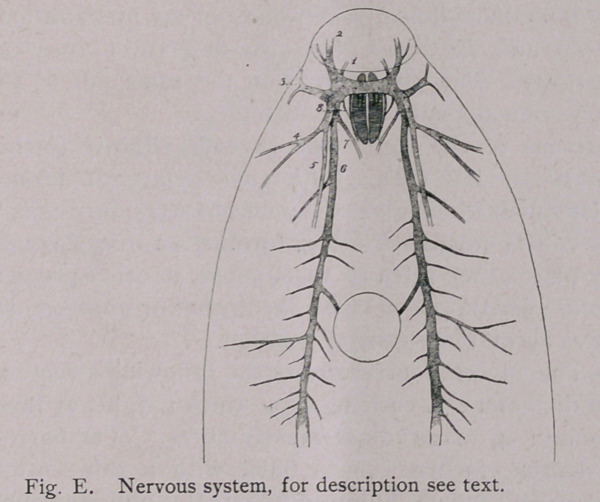


**Figs. F and G. f9:**